# Evolution of opto-electronic properties during film formation of complex semiconductors

**DOI:** 10.1038/srep45463

**Published:** 2017-04-04

**Authors:** M. D. Heinemann, R. Mainz, F. Österle, H. Rodriguez-Alvarez, D. Greiner, C. A. Kaufmann, T. Unold

**Affiliations:** 1 PVcomB, Helmholtz-Zentrum Berlin, Schwarzschildstraße 3, Berlin, 12489, Germany; 2 Helmholtz-Zentrum Berlin, Hahn-Meitner-Platz 1, Berlin, 14109, Germany

## Abstract

Optical and electrical properties of complex semiconducting alloys like Cu(In,Ga)Se_2_ (CIGS) are strongly influenced by the reaction pathways occurring during their deposition process. This makes it desirable to observe and control these properties in real-time during the deposition. Here we show for the first time the evolution of the band gap and the sub-band-gap defect absorption of CIGS thin film as well as surface roughness during a three-stage co-evaporation process by means of an optical analysis technique, based on white light reflectometry (WLR). By simultaneously recording structural information with *in-situ* energy dispersive X-ray diffraction and X-ray fluorescence we can directly correlate the evolution of opto-electronic material parameters with the structural properties of the film during growth. We find that the surface roughness and the sub-gap light absorption can be correlated with the phase evolution during the transformation from (In,Ga)_2_Se_3_ to Cu(In,Ga)Se_2_ by the incorporation of Cu into the film. Sub-bandgap light absorption is found to be influenced by the Cu-saturated growth phase and is lowered close to the points of stoichiometry, allowing for an advanced process design.

Chalcopyrite Cu(In,Ga)Se_2_ semiconductors have been among the drivers of thin film solar cell technology. The material exhibits excellent opto-electronic properties and can be deposited on a wide range of substrate materials such as soda-lime glass or flexible polyimide foil^1^. Laboratory champion solar cell efficiencies for this material have now reached 22.6%^2^ using a multi-stage co-evaporation technique. Until now record efficiencies have been achieved for significantly non-stoichiometric, Cu-poor material, which passed through a Cu-rich regime during growth and which includes a depth-dependent Ga-gradient leading to a larger band gap at the back and a minimum band gap closer to the front interface. In multi-stage growth processes, the overall elemental composition and gradients as well as structural phases evolve during the deposition, making process monitoring or control challenging.

Out of this reason typically rate monitoring^3^ in combination with end-point control by pyrometry^4^ or real-time laser light reflectometry is used^5^,^6^. More recently, the capability of real-time ellipsometry^7^ to monitor the evolution of film thickness and surface roughness has been demonstrated. White light reflectometry (WLR) is capable of providing comprehensive information on the microstructure^8^ and bandgap^9^ of semiconducting thin films. Recently, we have extended the WLR method to measure the sub-band gap absorption of CIGS thin films^10^.

However, the opto-electronic properties of the final material may depend strongly on the specific course of phase transitions during the complex deposition process. It is therefore highly desirable to gain access to the phase transitions and the opto-electronic properties during film growth to understand and to control the formation of the final properties.

Using *in situ* optical white light reflectometry (WLR) we show here for the first time how the band gap and sub-gap tail energy evolve during a multi-stage co-evaporation deposition process of CIGS. In combination with *in situ* X-ray diffraction^11^,^12^ we get access to structural, morphological and opto-electronic properties during the growth of CIGS. This enables the design of deposition routines leading to reduced sub-gap defect densities, necessary for further improvement of high-efficiency CIGS solar cells, and smooth CIGS films for the application in CIGS/Perovskite tandem cells^13^. The application of this method is not limited to the growth of chalcopyrite thin-films but could be adapted to kesterite, perovskite or any other compound semiconductor thin-film growth.

## Results

**Figure 1:**
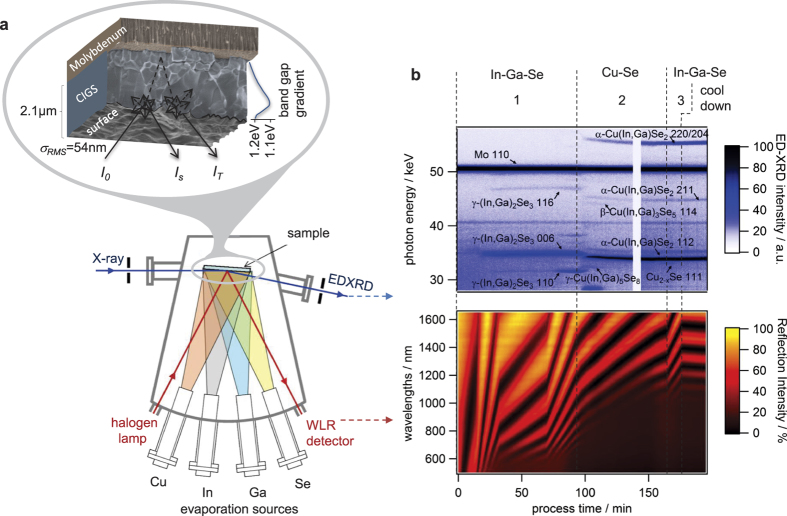
The experimental setup. (**a**) Schematic drawing of the PVD chamber and the WLR and ED-XRD measurement setup used to study the multi-stage co-evaporation process of Cu(In,Ga)Se_2_ films. An electron microscope image of the studied sample is shown on top, combined with its band gap gradient throughout the layer and a sketch of the specular and diffuse white light reflections. (**b**) Time evolution of the ED-XRD pattern and the WLR spectra during the three stages of the co-evaporation process.

The experimental setup of the real-time WLR and real-time EDXRD is schematically drawn in Fig. 1a. For the WLR measurement, broadband light from a halogen lamp is used to illuminate the center of the substrate and the specularly reflected light is recorded with a rate of 1 Hz (lower part of Fig. 1b). Simultaneously, EDXRD signals from the same sample position were recorded using polychromatic synchrotron light (upper part of Fig. 1b). (For more details see Methods).

**Figure 2:**
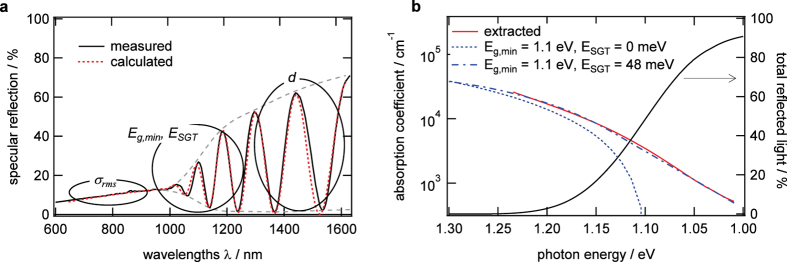
The WLR method. (**a**) An exemplary WLR spectrum, recorded at the end of the process after cool-down, shows the spectral regions which were used to deduct the surface roughness σ_RMS_, minimum band gap *E*_g,min_, sub-gap tail energy *E*_SGT_ and layer thickness *d*. The deviations of the fit to the experimental spectrum arise from the linear approximation of the refractive index (see method section). The total reflected light intensity can be calculated from the envelope of the interference fringes by correcting it for parasitic light absorption at the metallic back contact and diffuse scattering at the interfaces. This allows the extraction of the CIGS absorption coefficient, which is shown in (**b**) *E*_g,min_ and *E*_SGT_ are obtained by fitting the extracted absorption coefficient. The dashed blue lines are calculated absorption coefficients with two different sub-gap tail energies.

The spectral WLR intensities contain information on film roughness, film thickness, band-gap energy, and sub-gap light absorption. To illustrate the extraction of these values, a single WLR spectrum recorded at the end of the deposition process is depicted in Fig. 2a. In the shorter wavelength range up to 1000 nm, the photon energy is above the band-gap energy of *E*_g,min_ = 1.1 eV. *E*_g,min_ is defined as the smallest band gap within the graded absorber material and is therefore called the minimum band gap. Due to the high absorption coefficient of CIGS of α > 5 · 10^4^ cm^−1 ^14,15, photons within this wavelength range are nearly completely absorbed within the film and hence the reflectance is only influenced by the film roughness, σ_RMS_, and the refractive index at the surface^16^. In the upper wavelength range above 1100 nm, a fraction of the radiation, *I*_*T*_, penetrates the film, is reflected at the Mo back contact and interferes with radiation reflected at the surface, *I*_*S*_ (see inset in Fig. 1a). The shape of the resulting interference fringes envelope (dashed line in Fig. 2a) is mainly defined by the absorption coefficient, which can be extracted with equation (1) (method section) from the envelope as shown in Fig. 2b. By employing a self-consisting fitting procedure (method section) the values for the band gap energy *E*_g,min_ and for the sub-gap tail energy *E*_SGT_ can be extracted. The material properties resulting from the self-consistent fitting procedure, applied to the spectrum shown in Fig. 2a, compare very well to the results of independent *ex-situ* measurements. The root-mean-square surface roughness, σ_RMS_, obtained from the fitting procedure is 60 nm, compared to 54 nm obtained from atomic force microscope (AFM) measurement. The resulting minimum band gap *E*_g,min_ is 1.10 eV, compared to 1.11 eV calculated from elemental depth profiling by glow discharge optical spectroscopy (GDOES) and assuming a published relationship between the [Ga]/[In] + [Ga] content and the optical band gap in CIGS^14^. The sub-gap defect energy is calculated to be 48 meV, compared to 35 meV obtained from the low-energy exponential slope of a photoluminescence spectrum. The derived simplified refractive index (not shown) agrees well with the reported refractive index measured from Minoura *et al*.^15^ with a maximum deviation below 5% at all wavelengths.

**Figure 3:**
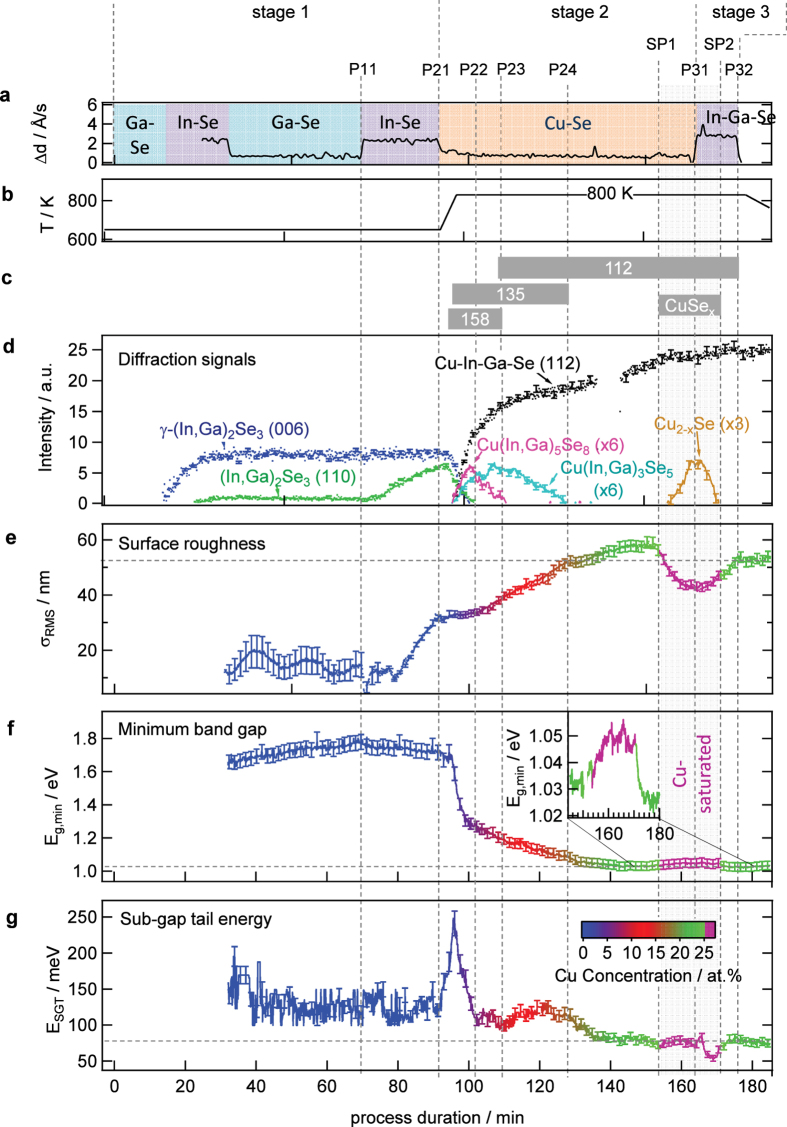
Evolution of the sample properties during the deposition process. (**a**) deposition rate during process stages, (**b**) substrate temperature, (**c**) crystalline phases detected by ED-XRD (**d**) diffraction signals, (**e**) surface roughness, (**f**) minimum band gap energy, (**g**) sub-gap tail energy. It should be noted that the band gap and the sub-gap tail energy depend on temperature. The missing data of the 112 diffraction signal in stage 2 was caused by an electron injection period of the synchrotron system.

The EDXRD and WLR signals recorded in real-time during CIGS growth are depicted in Fig. 1b as a function of process time. At the top of Fig. 1b the different deposition stages of the investigated CIGS co-evaporation process are indicated. The process starts with sequentially depositing In-Se and Ga-Se at a nominal substrate temperature of T_s_ = 620 K (stage 1), followed by Cu-Se deposition at T_s_ = 800 K (stage 2) until the CIGS film becomes Cu-rich up to [Cu]/([In] + [Ga]) = 1.09. Finally, In-Ga-Se is deposited at T_s_ = 800 K (stage 3) until the film becomes Cu-poor again ([Cu]/([In] + [Ga]) ≈ 0.9), as required for high-efficiency devices^17^,^18^. The relevance of the results for the understanding of the growth of high-quality solar cell absorbers is shown by a solar cell efficiency of 15% resulting from the analyzed CIGS absorber, despite non-optimized device design. The evolution of the layer properties during the deposition process are shown in Fig. 3. Deposition rates and temperature profile are shown in Fig. 3a and b, together with the EDXRD diffraction signals (Fig. 3d) and the surface roughness (Fig. 3e), the band-gap energy (Fig. 3f) and sub-gap tail energy (Fig. 3g), which are extracted from the WLR data. The evolution of these properties and their correlation will be described in detail in the following sections.

### Phase evolution and surface roughness

The evolution of the integral intensities of representative diffraction signals during the CIGS film deposition process is shown in Fig. 3d. The phase evolution from γ-(In,Ga)_2_Se_3_ (stage 1) to γ-Cu(In,Ga)_5_Se_8_ - > β-Cu(In,Ga)_3_Se_5_ - > α-Cu(In,Ga)Se_2_ - > α-Cu(In,Ga)Se_2_ + Cu_2−x_Se (stage 2) and back to α-Cu(In,Ga)Se_2_ (stage 3) follows the equilibrium phase diagram as reported before^19^,^20^. During the first part of stage 1, no clear diffraction signal is detectable, indicating that Ga-Se grows amorphous. During the first In-Se deposition of stage 1, In-rich (In,Ga)_2_Se_3_ grows with a preferred 006 orientation, while the film grows with a preferred 110 orientation during the second In-Se deposition of stage 1 (Fig. 3d). This change in orientation correlates with an increase in surface roughness (Fig. 3e), indicating the formation of larger and differently oriented grains.

At the beginning of the Cu-Se deposition (stage 2), the (In,Ga)_2_Se_3_ phase is transformed into Cu-In-Ga-Se, via the defect phases γ-Cu(In,Ga)_5_Se_8_ and β-Cu(In,Ga)_3_Se_5_ into the chalcopyrite α-Cu(In,Ga)Se_2_ phase. Note that the main Cu-In-Ga-Se signal (112) is present in all three phases - γ, β, and α^21^. The α phase can only be identified by the absence of signals belonging only to the β phase^21^. It is assumed that the α phase starts to form as soon as the signal of the β phase starts to decline. Initially, the surface roughness is not influenced by the transformation from (In,Ga)_2_Se_3_ into Cu-In-Ga-Se (Fig. 3d,e). This suggests that the film morphology does not change significantly during this transformation. Only after the β-Cu(In,Ga)_3_Se_5_ phase starts to decline, indicating the appearance of the α-Cu(In,Ga)Se_2_ phase, the surface roughness further increases. Inspection of the change of film roughness during stage 2 shows that there is a distinct change in this surface property at most points at which a structural phase appears or disappears in the EDXRD signal, e.g. point P23, P24 and SP1 in Fig. 3, which means that the phase transitions between the main structural phases can be observed optically during the deposition process.

When the Cu concentration exceeds Cu(In,Ga)Se_2_ stoichiometry ([Cu]/([In + Ga]) = 1), the Cu_2−x_Se (111) XRD signal rises indicating the segregation of Cu_2−x_Se at the surface (Fig. 3d, SP1)^19^,^20^. At the same point in time, the surface roughness decreases (Fig. 3e). It should be noted, that the Cu_2−x_Se segregation changes the refractive index of the surface which leads to the often observed increase of the diffuse reflectance^5^. However, the increase in the specular reflectance is larger than the increase in diffuse reflectance, proving that in fact the surface roughness is reduced. At the beginning of the evaporation of In-Ga-Se in stage 3, the Cu_2−x_Se signal declines and the surface roughness increases until the Cu_2−x_Se signal disappears, which is due to the transformation of Cu_2−x_Se into additional Cu(In,Ga)Se_2_. When passing the second point of stoichiometry (SP2) the course of the roughness exhibits a shoulder at a roughness value considerably smaller compared to the value at the first point of stoichiometry (SP1). A SEM cross-section image of the final CIGS layer is shown in Fig. 1a.

### Band gap evolution during chalcopyrite formation

During stage 1, the band gap *E*_g,min_ varies between 1.7 and 1.8 eV (Fig. 3g), which is close to the band gap of γ-In_2_Se_3_ of 1.8 eV at ambient temperatures^22^. This suggests that only little inter-diffusion within the Ga_2_Se_3_ and In_2_Se_3_ stacks takes place. Shortly after the beginning of Cu-Se co-evaporation in stage 2, *E*_g,min_ decreases quickly from ∼1.7 eV down to ∼1.3 eV. This decrease correlates with the first appearance of the Cu(In,Ga)_5_Se_8_ signal at an integral Cu concentration of ∼2.5 at.% (Fig. 3d). When the Cu(In,Ga)_5_Se_8_ signal starts decreasing at a Cu composition of around ∼5 at.%, the decrease in *E*_g,min_ slows down significantly. With further increasing Cu concentration, up until 20 at.%, the band gap decreases from 1.3 eV down to 1.03 eV and stays approximately constant beyond 22 at.% until Cu(In,Ga)Se_2_ stoichiometry is reached (SP1).

Besides the above mentioned dependence of the band gap on the Cu concentration, *E*_g,min_ is also influenced by the Ga concentration^22^, which is non-uniformly distributed throughout the film due to the different diffusion constants of Ga and In (see inset in Fig. 1a) [123]. According to diffusion models^21^,^23^ the [Ga]/([Ga] + [In]) ratio close to the surface is lowered during Cu-Se deposition in stage 2. This effect is confirmed by our real-time WLR data. The temperature-corrected *E*_g,min_ value (−0.16 meV/K) at the single phase compositions β-Cu(In,Ga)_3_Se_5_ (11 at.%) is 1.25 eV, which translates into a [Ga]/([Ga] + [In]) ratio of ∼0.1^24^,^25^, low compared to the integral composition of [Ga]/([Ga] + [In]) = 0.3 and also lower than the minimum [Ga]/([Ga] + [In]) ratio of ∼0.15 of the final film (obtained from elemental depth profiling).

**Figure 4:**
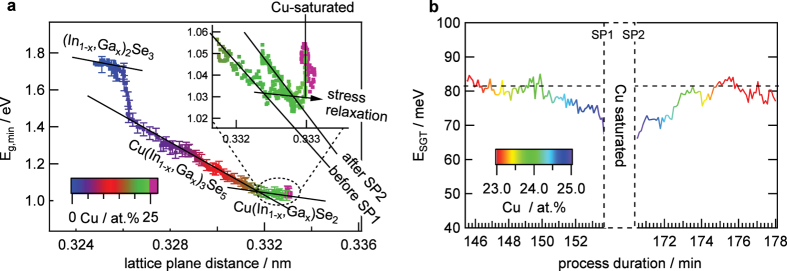
Correlation of optical and structural/compositional properties. (**a**) Correlation of the band gap energy with the 006 (In,Ga)_2_Se_3_ and 112 CIGS lattice spacing. The inset shows the region close to the points of stoichiometry. It should be noted, that due to the Ga gradient a small offset between the measured *E*_g,min_ and the band gap energy corresponding to a certain lattice plane distance may exist. (**b**) Correlation of the sub-gap tail-energy with the process duration and the Cu concentration.

In Fig. 4 the band gap *E*_g,min_ is plotted versus lattice plain distance of the 006 γ-(In,Ga)_2_Se_3_ and the 112 diffraction signal of the γ-Cu(In,Ga)_5_Se_8_, β-Cu(In,Ga)_3_Se_5_ and α-Cu(In,Ga)Se_2_ phase. The γ-Cu(In,Ga)_5_Se_8_ phase is the main phase for Cu concentrations between 3 and 6 at.% (Fig. 3c) and the observed band gap energy in this range is expected to be the band gap energy of the γ-Cu(In,Ga)_5_Se_8_ phase. For higher Cu concentration, the correlation of band gap and lattice plain distance follow a different but still linear relationship to each other until a Cu concentration of 18.5 at.%. Within this range of Cu concentrations the β-Cu(In,Ga)_3_Se_5_ phase is the main phase. At higher Cu concentrations the linear correlation again changes slope, indicating a new dominating crystal structure, which is the α-Cu(In,Ga)Se_2_ phase. This is in line with the disappearance of the β-Cu(In,Ga)_3_Se_5_ EDXRD signal at this Cu concentration (Fig. 3d). A similar trend was observed in ref. 24. Interestingly, another transition can be observed once the material becomes Cu saturated (SP1), as seen in the inset of Fig. 4a. At this point the band gap increases without changes in the lattice plain distance. This indicates that the increase of the optical band gap, which can also be seen in the inset of Fig. 3f, is due to a change in the CIGS defect composition, as it was speculated in ref. 26. When turning Cu-poor again in the third process stage the correlation starts again to follow a linear relationship, however with a slight offset to the previous linear slope. This can be explained by a relaxation of stress within the film. It was shown in ref. 27 that relaxation of lateral stress occurs by re-crystallization during the transition from Cu-poor to Cu-rich. However, it should be noted, that this stress relaxation occurs slightly prior to the Cu-saturated regime and that we cannot observe any influence of the relaxation on the optical band gap.

### Evolution of sub-gap tail-energy with Cu-concentration

The sub-gap tail energy *E*_SGT_ is obtained from the exponential tail within the absorption coefficient as shown in Fig. 2b. The origin of the observed tail could be due to disorder induced defect states or due to the existence of secondary phases with lower band gaps^24^. The evolution of *E*_SGT_ during the growth process is shown in Fig. 3f. It should be noted that the sub-gap tail energy increases linearly with increasing temperature^28^,^29^. In this study it is 0.05 meV/K.

During the co-evaporation of In-Se and Ga-Se in the first stage the tail energy remains constant. With the beginning of the transformation from the γ-(In,Ga)_2_Se_3_ phase into the γ-Cu(In,Ga)_5_Se_8_ and the β-Cu(In,Ga)_3_Se_5_ phase the tail energy peaks the moment all three phases exist at the same time and just before the minimum band gap starts to drop. Once the γ-(In,Ga)_2_Se_3_ phase has completely disappeared the tail energy levels off. It reaches a minimum once the γ-Cu(In,Ga)_5_Se_8_ phase has disappeared and the film has reached the Cu(In,Ga)_3_Se_5_ stoichiometry. After this point, the tail energy rises again. While the observed band gap is attributed to the β-Cu(In,Ga)_3_Se_5_ phase (Fig. 4a), the lower band gap of the α-Cu(In,Ga)Se_2_ phase likely leads to the observed increase in the tail energy. Once the β-Cu(In,Ga)_3_Se_5_ has fully disappeared, at a Cu concentration of around 20%, the band gap is solely attributed to the α-Cu(In,Ga)Se_2_ phase whose tail energy remains constant until a Cu concentration of 24%.

During the remaining process two additional minima of the tail energy can be observed, one at the first point of stoichiometry (SP1) and a second one around the second point of stoichiometry (SP2). The minimum around the SP2 is more pronounced, however, it occurs in the Cu-saturated regime during which the secondary Cu_2−x_Se phase exists. The tail energy in the Cu-poor regime is amplified in Fig. 4b. A reduced tail energy can be observed for a Cu concentration between 24 and 25 at.%. The closer the material comes to the point of stoichiometry the more its tail energy decreases. The dependency of the tail energy on the Cu concentration is similar before and after the Cu-saturated phase, with lower values after the Cu-saturated phase.

## Discussion

The good agreement between the *in-situ* obtained properties of the final layer with the independently *ex-situ* measured properties shows the ability of the WLR method to characterize the optical properties of a growing multi-crystalline CIGS layer in real-time. Additionally the correlation between these properties and the structural properties show that the crystallization process of CIGS, including phase evolution, grain orientation and grain growth can be observed indirectly with the help of the optical properties. Changes in the surface roughness were shown to indicate changes of the orientation or the appearance and disappearance of secondary phases.

During co-evaporation of the γ-(In,Ga)_2_Se_3_ precursor, an increasing 110 orientation of the γ-(In,Ga)_2_Se_3_ precursor is correlated with an increase of the surface roughness. It has been reported that the precursor orientation is correlated to the Se flux^30^ and since the precursor orientation influences the final orientation of the CIGS film and with it the device performance^30^,^31^, the observed correlation could be used to monitor the precursor quality. During stage 2, a continuous increase of the surface roughness can be observed. It is known that the average grain size increases with growing Cu content^17^,^32^, which could explain this increase of the surface roughness. Just before the point of stoichiometry (SP1) is reached, a reduction of the surface roughness was observed. This reduction of roughness could be due to the recrystallization, which occurs just before the observed formation of the Cu_2−x_Se secondary phase, as seen in the shift of the lattice plain distance in the inset of Fig. 4a. Thus it seems possible to use real-time WLR as an early indicator for the Cu_2−x_Se formation. The presence of Cu_2−x_Se was observed to further flatten the surface, since the surface roughness decreases with increasing Cu_2−x_Se concentration and vice versa. This indicates that Cu_2−x_Se preferably forms within the valleys of the CIGS surface. The surface roughness after the Cu-rich phase is still reduced compared to the roughness at the same Cu concentration before the Cu-rich phase. Hence, to achieve smooth films it is beneficial to keep the third stage as short as possible, because the roughness increases during this stage again.

The complete transformation of the γ-(In,Ga)_2_Se_3_ precursor into the CIGS phases can be very well observed by the evolution of the sub-gap tail energy *E*_*SGT.*_It should be noted, that the sub-gap tail energy is sensitive to secondary phases within the bulk only if their band gap energy is lower compared to the main phase present at that time. Shortly after the beginning of the evaporation of Cu (P21, Fig. 3), γ-Cu(In,Ga)_5_Se_8_ starts to form as a secondary phase. The lower band gap of this phase compared to the γ-(In,Ga)_2_Se_3_ precursor leads to the observed increase of *E*_*SGT*_. However, once it becomes the main phase, which is indicated by the drop of *E*_g,min_ (P22), *E*_*SGT*_ decreases again. At the point P23 *E*_*SGT*_ stops decreasing, because the β-Cu(In,Ga)_3_Se_5_ phase becomes the main phase while no other secondary phase with lower band gap energy exists at this point in time. Thus, *E*_*SGT*_is now determined by the amount of disorder and defects within the β-Cu(In,Ga)_3_Se_5_ material. At point P24 the β-Cu(In,Ga)_3_Se_5_ phase reaches stoichiometry (P24), leading to a minimum of *E*_*SGT*_, which can be explained by a reduced amount of disorder and defects. Beyond this point, the α-Cu(In,Ga)Se_2_ phase develops which has a lower bandgap compared to the β-Cu(In,Ga)_3_Se_5_ phase, leading again to an increase of *E*_*SGT*_. The disappearance of the β-Cu(In,Ga)_3_Se_5_ phase at P25 makes the α-Cu(In,Ga)Se_2_ the main phase at this point, leading to another reduction of the *E*_*SGT*_. Due to the absence of secondary phases with lower band gaps, the reduced sub-gap light absorption is now again defined by the disorder or defect density within that phase. Until a Cu concentration of 24 at.% is reached the *E*_SGT_ remains constant but drops for higher Cu concentrations. This can be explained by an improved crystal quality with lower disorder or defects for CIGS films with a Cu concentration close to the stoichiometric composition. At the first point of stoichiometry (SP1) and at the second point of stoichiometry (SP2) *E*_*SGT*_reaches its minimum. Also, the Cu-rich regime seems to expand the compositional width related to this minimum, because, when getting Cu poor again in stage 3, the range of Cu concentrations leading to a reduced defect density extends until 23.5 at.% and reduces the *E*_*SGT*_ overall by 5 meV within this range. The benefit of the *in-situ* determination of *E*_SGT_ is the possibility to terminate the process while the sub-gap defect energy is still low, but free of the Cu_2−x_Se phase, which is present in the Cu-rich growth regime. However, current state-of-the art devices employ Cu concentration below 24% (Cu/(Ga + In) < 0.93), to achieve a Cu deficiency at the surface. New surface passivation techniques such as KF^33^ or RbF^2^ post-deposition may allow the use of stoichiometric or close to stoichiometric absorbers in the future.

As it was shown in Fig. 4a, the course of the optical band gap plotted over the lattice constant is different for the different structural phases. The change of band gap with the lattice constant is lowest for the α-Cu(In,Ga)Se_2_ phase, which leads to the situation, that the minimum band gap at the end of stage 2 is close to the minimum band gap of the final film. This allows a direct control of the final minimum band gap by co-evaporation of Ga-Se in addition to Cu-Se in stage 2.

## Conclusion

The optical properties of CIGS have been studied *in-situ* and correlated to the structural properties during the deposition process. Several important results have been obtained. In particular it was shown that secondary phases can be detected by changes of the surface roughness and the sub-gap tail energy, allowing an easy accessible insight into the film formation. This could also become interesting for other complex compounds such as kesterites (Cu_2_(Zn,Sn)S_4_) or perovskites. Further, it was found that the sub-gap defect absorption is lowest for Cu-rich films and reduced for Cu concentration between 24 and 25 at.%. These findings allow an advanced process design with the aim to reduce the sub-gap defect states and also to *in-situ* control the minimum band gap. New tandem applications of the CIGS layers could benefit from the insights gained regarding the evolution of the surface roughness, which was shown to depend on the precursor orientation, the Cu diffusion and the Cu-rich phase.

## Methods

### WLR setup

Broadband light (300–1700 nm) from a halogen lamp is used to illuminate the center of the substrate. The specularly reflected light is detected with a Si-CCD and an InGaAs diode array at a rate of 1 Hz.

### WLR data evaluation

To obtain layer thickness *d*, surface roughness σ_rms_, and absorption coefficient *α*(*λ*) by fitting the specular reflection spectrum (Fig. 2a), a sequential fitting procedure was developed enabling an extraction of material parameters as required for the application of the method as a robust and reliable process control. As the refractive index of the growing layer is not known at any point during the process, the fitting procedure starts with literature values of the refractive index *n*_0._ First, the part of the spectrum less affected by the absorption coefficient, the interference-free region, is described with the help of the scalar scattering theory to extract the surface roughness:


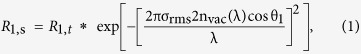


with *R*_1,s_describing the intensity of the specular reflected light from the CIGS surface, *R*_1,*t*_ the intensity of the total reflected light from the CIGS surface, ***σ***_*rms*_ the root mean square (RMS) surface roughness, *n*_*vac*_(*λ*)the refractive index of the vacuum, ***θ***_1_ is the incoming angle to the substrate normal. The second part is the low-energy region which is almost free of absorption losses. In this region the interference fringes allow the extraction of the layer thickness. With these two properties and by neglecting multiple reflections within the absorber layer, it is possible to extract the absorption coefficient from the amplitude *A* of the envelope of the interferences (see Fig. 2a):


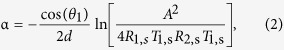


with *T*_1,s_ being the specular transmission at the CIGS/vacuum interface and *R*_2,s_ the specular reflection at the Mo back contact. After calculating the absorption coefficient, the whole spectrum is fitted with a parameterized refractive index *n* as the fitting parameter. The whole procedure is repeated until the fit quality does not further improve, leading to a self-consistent solution for all parameters, including an approximated refractive index.

### Uncertainties

To extract the band gap energy and sub-gap tail energy from the course of *α*(*λ*) we have to consider that the Ga distribution – and consequently the band gap energy – shows a depth gradient^34^. The absorption coefficient for the complete CIGS layer can be approximated by a sum of *m* layers with different direct band gap energies but identical prefactors, *B*, and sub-gap tail energies, *E*_SGT_. Thus we modified the formula given in ref. 35 for graded band gaps to:


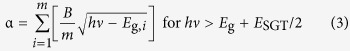



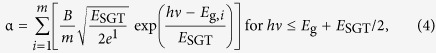


with *E*_g,*i*_ being the band gap energy of the *i*-th layer (with *E*_g,1_ = *E*_g,min_ and *E*_g,m_ = *E*_g,min_ + d*E*_g_, where d*E*_g_ is the difference between the smallest and the highest band gap energy). Each layer is weighted by 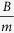, since the influence of the gallium concentration on *B* is negligible^15^. The distribution of band gaps of a typical CIGS absorber can be well described with a simple square like distribution, whose width increases over time due to the slow diffusion of Ga compared to In during the 2^nd^ stage of the deposition process. The developing band gap gradient, Δ*E*_g_, cannot be obtained from the fit to the absorption coefficient since the layers with the lowest band gap energy *E*_g,min_ dominate the transmission through the film (Fig. 2b). Within this model the gallium gradient is assumed to be linear over the sample depth with a slope increasing linearly over time from zero at the beginning of stage 2 until 0.075 eV/μm at the end of stage 2. The evolution of the CIGS 112 XRD peak width allows an approximation, but still an error of 40% is assumed. Fortunately the Ga gradient has only little influence on the sub-gap tail energy, an error of 40% of the Ga gradient induces a relative error of 0.4% to the sub-gap tail energy. The relative error of the minimum band gap energy is increased by 2.2%. The main source of error comes from the simplified refractive index. Assuming a relative error of 5% of the refractive index, the relative error of the layer thickness becomes 5%, of the surface roughness 3.4%, of the calculated minimum band gap only 0.3%, but the error of the sub-gap defect energy becomes 7.4%.

### EDXRD

*In-situ* EDXRD measurements were performed at the EDDY beamline of the BESSY II synchrotron facility with a two detector setup, as described in ref. 27. The accessible X-ray energy range was 6 to about 80 keV and the diffraction angle was fixed at 2***θ*** = 6.301° ± 0.002° for the first detector and 9.722°° ± 0.002° for the second detector. Every 10 s one X-ray spectrum was recorded. The information depth as defined in the supporting information of ref. 27 is 0.69 μm at the energy of the 112 reflex of CIGS in the second detector, which was used for the calculation of the lattice plain distance in Fig. 4a. To obtain the area and the energy of the XRD peaks they were fitted with a Gaussian function. The lattice parameters, *d*_*hkl*_, were calculated with the Bragg equation:


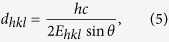


with *E*_*hkl*_ being energy positions of the recorded diffraction lines.

The Cu concentration at each point in time was calculated from the real-time Cu-K***α*** fluorescence data combined with the assumption that Cu_2−x_Se segregation starts at a Cu concentration of 25 at.% and that the Cu-Se deposition rate is constant during stage 2. It should be noted that Cu_2−x_Se segregation was also reported to occur at a Cu concentration of 24.5 at.%^36^.

## Additional Information

**How to cite this article:** Heinemann, M.D. *et al*. Evolution of opto-electronic properties during film formation of complex semiconductors. *Sci. Rep.* **7**, 45463; doi: 10.1038/srep45463 (2017).

**Publisher's note:** Springer Nature remains neutral with regard to jurisdictional claims in published maps and institutional affiliations.
